# Porcine Reproductive and Respiratory Syndrome Virus Antagonizes PCSK9’s Antiviral Effect via Nsp11 Endoribonuclease Activity

**DOI:** 10.3390/v12060655

**Published:** 2020-06-17

**Authors:** Yujiao Zhang, Fei Gao, Liwei Li, Kuan Zhao, Shan Jiang, Yifeng Jiang, Lingxue Yu, Yanjun Zhou, Changlong Liu, Guangzhi Tong

**Affiliations:** 1 Shanghai Veterinary Research Institute, Chinese Academy of Agricultural Sciences, Shanghai 200241, China; yujiaozhang1028@163.com (Y.Z.); feigao@shvri.ac.cn (F.G.); liliwei@shvri.ac.cn (L.L.); zhaokuan519@126.com (K.Z.); jiangshan_1995@163.com (S.J.); jiangyifeng@shvri.ac.cn (Y.J.); yulingxue@shvri.ac.cn (L.Y.); yjzhou@shvri.ac.cn (Y.Z.); 2 Jiangsu Co-Innovation Center for the Prevention and Control of Important Animal Infectious Disease and Zoonosis, Yangzhou University, Yangzhou 225009, China

**Keywords:** porcine reproductive and respiratory syndrome virus, PCSK9, CD163, nsp11, lysosome, endoribonuclease activity

## Abstract

Porcine reproductive and respiratory syndrome virus (PRRSV) is one of the most important pathogens in the swine industry worldwide. Our previous study had indicated that proprotein convertase subtilisin/kexin type 9 (PCSK9) was a responsive gene in porcine alveolar macrophages (PAMs) upon PRRSV infection. However, whether PCSK9 impacts the PRRSV replication and how the PRRSV modulates host PCSK9 remains elusive. Here, we demonstrated that PCSK9 protein suppressed the replication of both type-1 and type-2 PRRSV species. More specifically, the C-terminal domain of PCSK9 was responsible for the antiviral activity. Besides, we showed that PCSK9 inhibited PRRSV replication by targeting the virus receptor CD163 for degradation through the lysosome. In turn, PRRSV could down-regulate the expression of PCSK9 in both PAMs and MARC-145 cells. By screening the nonstructural proteins (nsps) of PRRSV, we showed that nsp11 could antagonize PCSK9’s antiviral activity. Furthermore, mutagenic analyses of PRRSV nsp11 revealed that the endoribonuclease activity of nsp11 was critical for antagonizing the antiviral effect of PCSK9. Collectively, our data provide further insights into the interaction between PRRSV and the cell host and offer a new potential target for the antiviral therapy of PRRSV.

## 1. Introduction

Porcine reproductive and respiratory syndrome (PRRS) has been a major disease affecting the swine industry worldwide since it was first reported in the USA in the late 1980s [1,2]. The causative agent PRRS virus (PRRSV) strains—which were isolated from two continents, Western Europe (type-1) and North America (type-2)—are tremendously different, with merely 55–70% nucleotide identity [2,3,4,5]. PRRSV is an enveloped, positive-sense, single-stranded RNA virus classified in the Arteriviridae family within the Nidovirales order [6,7,8]. The PRRSV genome is approximate 15 kb in length and encodes 11 known open reading frames (ORFs) [9,10,11]. During PRRSV infection, the replicase-associated polyproteins pp1a and pp1ab encoded by the ORF1a and ORF1b are cleaved by its papain-like protease nsp2 (nonstructural protein 2) and 3C-like protease nsp4 into more than 14 nonstructural proteins [12,13]. Eight structural proteins including glycoprotein 2 (GP2), envelope protein (E), GP3, GP4, ORF5a protein, GP5, membrane protein (M), and nucleocapsid protein (N) are encoded by ORF2, 2a, 3, 4, 5a, 5, 6, and 7, respectively [2,10,11,12].

The immune-modulatory roles of PRRSV nsps have drawn much attention. Accumulating evidence suggests that PRRSV nsp1α/β, nsp2, nsp4, nsp5, nsp7, nsp9, nsp10, and nsp11 have immunomodulatory functions [12,14]. Among those PRRSV nsps, nsp11 has endoribonuclease activity and is involved in multiple steps of the arterivirus replicative cycle [6,12]. Nsp11 contains a nidovirus uridylate-specific endoribonuclease domain (NendoU), which is indispensable for arterivirus replication [15,16,17]. The 129/144/173 site and major dimerization site (Ser74 and Phe76) of nsp11 are related to the endoribonuclease activity, and the inactivation of the endoribonuclease activity of nsp11 leads to the failure of the suppression of IFN-β production [18]. Nsp11 can block the phosphorylation of both IRF3 and IκB, resulting in the suppression of IFN production [19,20]. More recent studies reveal that the nsp11 of PRRSV antagonizes interferon signaling by inducing STAT2 degradation or targeting IRF9 [21,22]. In addition to regulating the cell host innate immune response through endoribonuclease activity, nsp11 can also inhibit NF-κB signaling through its deubiquitinating activity [23].

PRRSV infection leads to poor innate and adaptive immune responses associated with immune modulation and incomplete viral clearance in most of the pigs. The ability of PRRSV infection to suppress IFNα secretion from macrophages and plasmacytoid dendritic cells is widely understood. Infection with PRRSV stimulates an antibody response by 7–9 days post-infection but with no evidence of protection against PRRSV infection, and serum neutralizing antibodies appear only later [2]. The commercially available vaccines have limited cross-protective efficacy against heterologous infections. Therefore, understanding the interaction of PRRSV and the cell host can offer potential targets for antiviral therapies. Numerous host factors have been reported as either facilitating or suppressing virus infection and/or replication. These factors encompass cell surface receptors for PRRSV infection such as CD163, sialoadhesin, heparin sulfate, vimentin, CD151, and CD209 [24] and other cellular factors such as MYH9 [25], heparinase [26], CH25H [27,28], nucleoporin 62 [29], annexin A2 [30], ZAP [31], DHX36 [32], and so forth. Likewise, studies from our laboratory show that membrane proteins including MOV10, galectin-1, and galectin-3 can influence the replication of PRRSV [33,34,35].

Proprotein convertase subtilisin/kexin type 9 (PCSK9) is an enzyme encoded by the PCSK9 gene in humans and belongs to a family of nine subtilisin-like serine proteases, which is involved in the proteolytic maturation of different proteins like hormones and cytokines [36,37]. PCSK9 plays a crucial role in plasma cholesterol metabolism in that it regulates low-density lipoprotein receptor (LDLR) levels by increasing LDLR degradation [38,39,40,41]. Human PCSK9 is a 692-amino acid glycoprotein, synthesized as a 72 kDa soluble zymogen (proPCSK9). The proPCSK9 releases a 14 kDa peptide by an autocatalytic process. The released protein remains attached to the mature protein to inactivate the catalytic domain [37,42]. In addition to the major role of PCSK9 in the regulation of LDLR, it has been reported that PCSK9 can impede hepatitis C virus (HCV) infection by modulating the levels of the HCV receptors LDLR and CD81 on the liver cell surface [43]. Apart from the antiviral effect of PCSK9 on HCV, serum PCSK9 levels are increased in HIV-positive individuals and are associated with abnormal coronary endothelial function [44].

In the current study, we aimed to explore the relationship between PCSK9 and PRRSV replication. We demonstrated that PCSK9 could inhibit PRRSV replication by targeting the virus receptor CD163 for degradation through the lysosome. Moreover, PRRSV down-regulated the expression of PCSK9 in the late stages during PRRSV infection. Specifically, nsp11 antagonized PCSK9’s antiviral activity through its endoribonuclease activity. The data improve our understanding of the mechanism of PRRSV interference with the host factor.

## 2. Materials and Methods

### 2.1. Cell Culture and Viruses

MARC-145, Hela, and HEK-293T cells (ATCC, America) were maintained in DMEM (Gibco, Shanghai, China) supplemented with 10% heat-inactivated FBS (Gibco, Australia) at 37 °C under 5% CO_2_ in a humidified incubator. Porcine alveolar macrophages (PAMs) were prepared from the lung lavage fluid of 6-week-old healthy piglets free of PRRSV and were cultured in RPMI 1640 (Sigma, Shanghai, China) containing 10% heat-inactivated FBS (Gibco, Australia) at 37 °C under 5% CO_2_. The HP-PRRSV strain HuN4 is a type-2 (North American) PRRSV that was isolated in China at the end of 2006 [45]. The highly pathogenic PRRSV HuN4 (GenBank accession no. EF635006), the attenuated vaccine virus HuN4-F112 [46], the classic type-2 strain APRRS (GenBank accession no. GQ330474), and the classic type-1 strain Lelystad (GenBank accession No. GQ461593) are stored in our laboratory. The titer of PRRSV was determined in MARC-145 cells.

### 2.2. Plasmid Construction

The coding sequence of wild-type porcine PCSK9 (XM_005653438.2) was synthesized and cloned into the pCAGGS vector to generate the Flag-tagged expression plasmid pCAGGS-PCSK9-Flag. The Flag tag was followed by the termination codon. The PCSK9-151-415-Flag and PCSK9-463-705-Flag plasmids were constructed by PCR amplification from the wild-type pCAGGS-PCSK9-Flag plasmid. The mutant vectors (pCAGGS-PCSK9-Q150A, pCAGGS-PCSK9-D197A, pCAGGS-PCSK9-H237A, pCAGGS-PCSK9-N328A, pCAGGS-PCSK9-S397A, pCAGGS-HA-Nsp11-C112A, pCAGGS-HA-Nsp11-H129A, pCAGGS-HA-Nsp11-H144A, pCAGGS-HA-Nsp11-K173A, pCAGGS-HA-Nsp11-C112K173A, and pCAGGS-HA-Nsp11-H129H144A) were generated from the wild-type pCAGGS-PCSK9-Flag and pCAGGS-HA-Nsp11 by site-directed mutagenesis. Pig CD163 was amplified from PAM cells’ cDNA and cloned into pCAGGS with the HA tag. The promoter sequence (−800~+55 of the transcription start site) of the porcine PCSK9 gene (Gene ID: 100620501) and the promoter sequence (−296~+52 of the transcription start site) of the porcine IFNB1 gene (Gene ID: 445459) were constructed by PCR amplification from the PAM cells’ genome and cloned into the pGL3-Basic vector. All the primers used for plasmid construction are listed in Table 1.

### 2.3. Reagents and Antibodies

The monoclonal antibody against the PRRSV N protein was a kind present from Dr. Ying Fang (Department of Animal Sciences and Industry, Kansas State University, Manhattan). Monoclonal antibodies against PRRSV nsp2, PCSK9, and CD163 were produced and stored by our lab. Goat anti-mouse IgG (H + L) antibody conjugated with Alexa Fluor 488 (1:800) was purchased from Abcam (Abcam, Shanghai, China). MG132 and CQ were purchased from Sigma (Sigma, Shanghai, China). DMSO was purchased from MP Biomedicals (MP Biomedicals, Shanghai, China). Lipofectamine^®^3000 Transfection Kits were purchased from Invitrogen (Invitrogen, Shanghai, China). Dual Luciferase Reporter Assay Kits were purchased from Vazyme Biotechnology (Vazyme Biotechnology, Nanjing, China). Monoclonal Anti-HA-Agarose antibody produced in mice was purchased from Sigma (Sigma, Shanghai, China). PrimeSTAR^®^ HS DNA Polymerase with GC Buffer was purchased from Takara (Takara, Dalian, China). DAPI was purchased from Beyotime (Beyotime, Shanghai, China).

### 2.4. Multi-Step Growth Curve of Virus

MARC-145 cells cultured in 6-well plates were transfected with either pCAGGS-PCSK9-Flag or the empty vector pCAGGS in triplicate and then infected with PRRSV at an MOI (Multiplicity of infection) of 0.1 at 36 h after transfection. Then, 250 μL of supernatant was collected at different time points post infection, followed by the addition of 250 μL of fresh DMEM with 2% FBS (Gibco, Australia). One hundred microliters of supernatant was used for the TCID50 assay.

### 2.5. TCID50 Assay for PRRSV

MARC-145 cells were seeded in 96-well plates and then infected with serial 10-fold dilutions of PRRSV samples in eight replicates. Ninety-six hours later, the virus titers were calculated based on the Reed–Muench method.

### 2.6. Indirect Immunofluorescence Assay

Cells were washed with cold PBS 3 times before being fixed with cold methanol for 10 min, and then, the cells were incubated with 5% BSA at 37 °C for 1 h prior to primary antibody (anti-Mouse PRRSV nsp2 monoclonal antibody, 1:800 or rabbit anti-Flag monoclonal antibody, 1:2000, Sigma, Shanghai, China) incubation for 1.5 h. The cells were washed in PBS 4 times, incubated with goat anti-mouse IgG (H + L) antibody conjugated with Alexa Fluor 488 (Abcam, Shanghai, China, 1:800) or goat anti-rabbit IgG (H + L) antibody labeled with Alexa Fluor 596 (Life Technologies, Shanghai, China, 1:800) for 1 h, and washed 4 times with PBS. The cells were counterstained with 5 μg/mL of 4′, 6′-diamidino-2-phenylindole (DAPI) (Beyotime, Shanghai, China) for 10 min. Images were taken using a microscope equipped with a monochrome EMCCD camera (Zeiss, Germany).

### 2.7. RNA Extraction and RT-qPCR Assay

For virus copy number detection, total RNA was extracted from cultured cells with the RNeasy^®^ Mini Kit (QIAGEN, Hilden, Germany), and the RNA in the supernatant was extracted with the QIAamp Viral RNA Mini Kit (QIAGEN, Hilden, Germany). The RNA was then reverse transcribed into cDNA with a reverse transcriptase mix (Takara, Dalian, China). For PAM RNA extraction, cells were washed using PBS three times before RNA extraction. To each sample was added 333 uL of TRIzol (Thermo Fisher Scientific, Shanghai, China). Three samples were merged into one sample, and the total RNA was extracted and then reverse transcribed into cDNA with reverse transcriptase (Thermo Fisher Scientific, Shanghai, China). Quantitative real-time PCR experiments were performed in triplicate. The relative level of mRNA expression was normalized to that of GAPDH. Absolute quantitative mRNA levels were calculated using standard curves.

### 2.8. Co-Immunoprecipitation (Co-IP)

Co-IP was performed as described previously [33]. Briefly, HEK-293T cells were co-transfected with the indicated plasmid using Lipofectamine 3000 Transfection Kits (Invitrogen, Shanghai, China). Twenty-four hours after transfection, the lysates were collected with the IP lysis buffer (Thermo Fisher Scientific, Shanghai, China), and then 15 μL of Agarose beads with monoclonal antibody against HA or Flag (Sigma, Shanghai, China) were added to each sample and incubated at 4 °C for 6 h with rotation. Then, the beads were pelleted and washed 5 times with IP lysis buffer. Finally, the proteins were dissolved in 50 μL of IP lysis buffer.

### 2.9. Confocal Microscopy

HeLa cells were seeded on sterile glass coverslips in 6-well plates then transfected with pCAGGS-PCSK9-Flag and/or pCAGGS-CD163-HA. Twenty-four hours post-transfection (hpt), the cells were washed with PBS twice, fixed with 550 uL of cold methyl alcohol for 10 min, and blocked with 5% bovine serum albumin (Shenggong, Shanghai, China) for 30 min. The transfected cells were washed with PBS three times, incubated with mouse anti-HA Mab (Sigma, Shanghai, China, 1:2000) and rabbit anti-Flag Mab (Sigma, Shanghai, China, 1:2000) for 1 h at 37 °C, and washed three times with PBS. The cells were then incubated at 37 °C for 1 h with donkey anti-mouse IgG (H + L) antibody conjugated with Alexa Fluor 596 (Life Technologies, Shanghai, China, 1:800) and goat anti-rabbit IgG (H + L) antibody labeled with Alexa Fluor 488 (Life Technologies, Shanghai, China, 1:800). The cells were counterstained with 5 μg/mL of 4′, 6′-diamidino-2-phenylindole (DAPI, Beyotime, Shanghai, China) for 10 min. Images were taken using a Zeiss confocal system (Zeiss, Germany).

### 2.10. Western Blotting

Cell lysates were prepared using RIPA buffer (Thermo Fisher Scientific, Shanghai, China) or IP Lysis buffer (Thermo Fisher Scientific, Shanghai, China) supplemented with protease inhibitor and phosphatase inhibitor (Bimake, Shanghai, China). Equal amounts of cell lysates were fractionated by SDS-PAGE and then blotted onto nitrocellulose membranes. Membranes were blocked for 1 h at room temperature (RT) with 5% nonfat milk and then probed with specific primary antibodies for 2 h at RT. After being washed with TBST (10 min for each wash), the membranes were incubated with either horseradish peroxidase (HRP)-conjugated goat anti-rabbit or goat anti-mouse antibody (Proteintech, Wuhan, China, 1:6000;) for 1 h at RT. After the membranes were washed three times, signals were raised using an ECL kit (Thermo fisher, Shanghai, China) and detected with a Tanon-5200 automatic chemiluminescence image analysis system. The primary antibodies used were as follows: mouse anti-HA (Sigma, Shanghai, China, 1:6000), rabbit anti-Flag (Sigma, Shanghai, China, 1:6000), mouse anti-β-actin produced (Sigma, Shanghai, China, 1:6000), N polyclonal antibody produced in mouse (1:500; reserved in laboratory), mouse anti-PCSK9 (Detai Biotechnology, Nanjing, China, 1:1000), and mouse anti-MYC (Cell Signal Technology, Shanghai, China, 1:1000;).

### 2.11. Reporter Assay

HEK-293T cells were seeded in 12-well plates and transfected with related plasmids when the cells reached 70% confluence, and they were transfected with a mixture of IFN-β-luc and pRL-TK-Renilla luciferase plasmids and appropriate control or protein-expressing plasmid(s). Twenty-four hours after transfection, the cells were treated with regent poly (I:C) (Invivogen, Shanghai, China) for the IFN stimulation and then collected at 12 h after treatment. Reporter gene activity was determined by the normalization of firefly luciferase activity against Renilla luciferase activity.

### 2.12. Bioinformatics Prediction

The I-TASSER online service tool was used to predict the 3D structure of porcine PCSK9, referring to the human PCSK9 protein (Protein Data Bank, ID: 2P4E). The 3D structure of porcine PCSK9 was visualized using the VMD software (Version 1.9.4, IL, USA).

### 2.13. Statistical Analyses

All data were analyzed with GraphPad Prism 5 (GraphPad, San Diego, CA, USA) and are provided as the mean ± SEM unless otherwise specified in figure legends. Statistical analyses were performed using the unpaired two-tailed Student’s t-test. Differences between groups were considered statistically significant when the *p* value was less than 0.05 (*, *p* ≤ 0.05; **, *p* ≤ 0.01; ***, *p* ≤ 0.001). The gray values from Western blotting were calculated using the Image J software (https://imagej.nih.gov/ij/).

## 3. Results

### 3.1. PCSK9 Inhibits the Replication of Both Type-1 and Type-2 PRRSV Species

**Figure 1:**
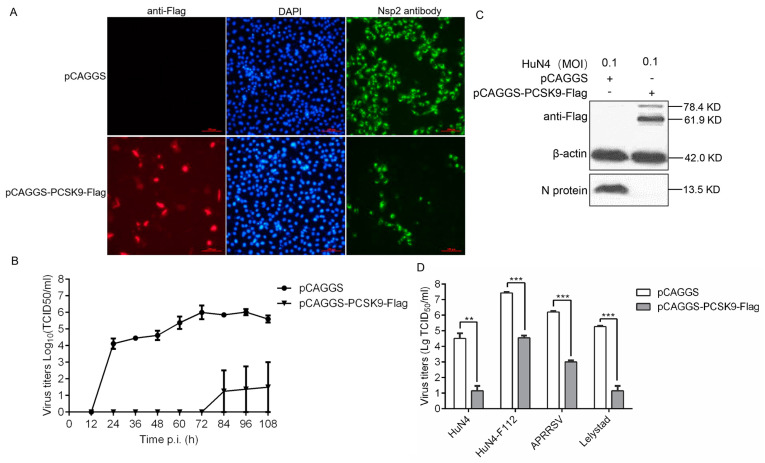
PCSK9 inhibits porcine reproductive and respiratory syndrome virus (PRRSV) replication. MARC-145 cells were transfected with 2.5 μg of either pCAGGS-PCSK9-flag or the empty vector pCAGGS as a negative control. At 36 hours post-transfection (hpt), the cells were treated as follows: (**A**) The cells were infected with the PRRSV HuN4 strain (MOI = 0.1). An immunofluorescence assay was performed to detect the PRRSV nsp2 protein (green) and PCSK9 (red) to assess the replication of the virus. The cells were counterstained with DAPI. Representative images from triplicate experiments are shown. Scale bar: 100 μm (**B**) The cells were infected with the HuN4 PRRSV (MOI = 0.1). The supernatants were collected at the indicated time points after infection, and the virus titers were determined on MARC-145 cells as the TCID50. (**C**) The cells were infected with HuN4 (MOI = 0.1). PCSK9 protein and PRRSV N protein were analyzed by Western blotting (WB) at 36 hpi (hours post infection). (**D**) The cells were infected with the type-2 PRRSV strains APRRSV, HuN4, and F112 and type-1 PRRSV strain Lelystad (MOI = 0.1), respectively. At 48 hpi, the virus titers in the supernatants were determined as the TCID50 on MARC-145 cells. Error bar: mean ± SEM; **, *p* ≤ 0.01; ***, *p* ≤ 0.001.

In our previous study, we performed a liquid chromatography-tandem mass spectrometry (LS-MS/MS) analysis of PAMs infected with or without the PRRSV strain HuN4-eGFP. The LS-MS/MS result showed that the PCSK9 expression level increased significantly upon PRRSV strain HuN4-eGFP infection in PAMs compared to in the mock infected PAMs [47]. However, the function of PCSK9 in PRRSV replication is not yet defined. To this end, we overexpressed PCSK9 in MARC-145 cells by transfecting a vector expressing porcine PCSK9 to evaluate the effect of PCSK9 on PRRSV replication. Firstly, the PCSK9-transfected MARC-145 cells were infected with the highly pathogenic PRRSV strain HuN4, and then, the expression of the PRRSV nsp2 protein was analyzed by immunofluorescence. As shown in Figure 1A, the fluorescence signal was significantly lower in pCAGGS-PCSK9-Flag-transfected cells compared to that in the empty vector pCAGGS-transfected cells. In addition, we collected virus-containing supernatant after HuN4 strain infection from pCAGGS-PCSK9-Flag- and pCAGGS-transfected MARC-145 cells at different time points. Compared to those in control, the virus titers from the PSCK9-overexpressing cells were much lower (Figure 1B). We further confirmed this finding by investigating the expression level of the PCSK9 protein and PRRSV N protein using Western blotting (WB) (Figure 1C). Taken together, these data indicated that PCSK9 inhibited PRRSV replication in vitro.

Then, we reasoned that PCSK9 could suppress not only type-2 PRRSV replication but also type-1 PRRSV replication. Therefore, we infected PCSK9-overexpressing cells with the type-1 PRRSV strain Lelystad as well as the type-2 PRRSV strains APRRSV, HuN4, and F112 and collected the supernatants from the infected cells. The virus titers decreased in PCSK9-overexpressing cells compared to those in the control cells for both types of PRRSV (Figure 1D). This result demonstrated that the ectopic expression of PCSK9 led to the suppression of both type-1 and type-2 PRRSV replication.

### 3.2. The C-Terminal Domain of PCSK9 Has Antiviral Activity

**Figure 2:**
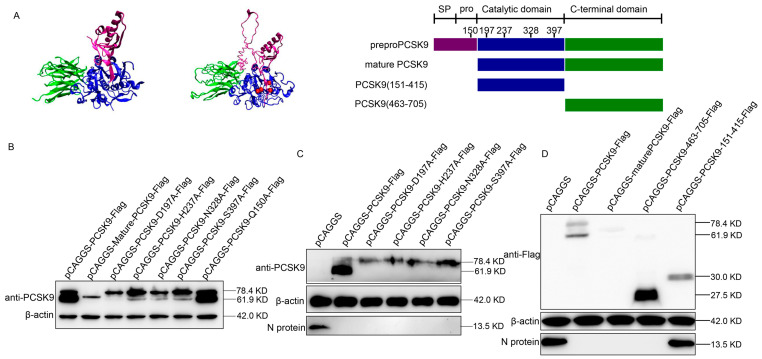
The C-terminal domain of PCSK9 has antiviral activity. (**A**) Left: 3D structure of human PCSK9 protein; middle: predicted 3D structure of porcine PCSK9 protein using program I-TASSER; red sphere: residues D197, H237, N328, and S397; purple: SP (signal peptide) and prodomain; blue: catalytic domain; green: C-terminal domain; right: domain organization and engineering of single residue mutant and truncation constructs of PCSK9. (**B**) MARC-145 cells were transfected with either mutated PCSK9 (D197A, H237A, N328A, S397A, and Q150A) or the empty vector pCAGGS. At 36 hpt, the expression of PCSK9 was analyzed by WB. (**C**) MARC-145 cells were transfected with either mutated PCSK9 or empty vector pCAGGS. At 36 hpt, the cells were infected with HuN4 PRRSV (MOI = 0.1). At 36 hpi, cell lysates were analyzed by WB. (**D**) MARC-145 cells were transfected with PCSK9-Flag, mature-PCSK9-Flag, PCSK9-151-415-Flag, and PCSK9-463-705-Flag, respectively. The cells were then infected with HuN4 PRRSV (MOI = 0.1), and the cell lysates were analyzed by WB at 36 hpi.

The human PCSK9 protein consists of a signal sequence followed by a pro-domain, a catalytic domain, and a C-terminal domain. The PCSK9 protein is synthesized as an inactive pro-enzyme and contains a triad of residues (Asp-186, His-226, and Ser-386) that are required for catalytic activity [42,48]. Human PCSK9 has been well studied since it is a key player in plasma cholesterol metabolism. However, there is limited information available about the porcine PCSK9 protein. Thus, we compared the human and porcine PCSK9 protein structures and found that porcine PCSK9 contained similar domains to human PCSK9 (Figure 2A). We also obtained the residues that were possibly responsible for porcine PCSK9 protein maturation (Figure 2A). To confirm if those residues were involved in PCSK9 pre-protein catalysis, we generated a set of single amino acid-mutated variants of the PCSK9 protein (Figure 2A). The WB result showed that residues 197, 237, 328, and 397 were required for PCSK9 protein maturation, whereas residue 150 was not necessary (Figure 2B).

We speculated that mature PCSK9, but not immature PCSK9, possessed antiviral activity. Therefore, we examined the antiviral activity of these mutated PCSK9 proteins by overexpressing these mutants in MARC-145 cells, followed by PRRSV strain HuN4 infection. Interestingly, the WB analysis of the PCSK9 protein and PRRSV N protein showed that a single residue mutation in PCSK9 could not affect the antiviral activity of PCSK9 (Figure 2C). Furthermore, to determine which domain of PCSK9 protein is crucial for the antiviral activity, we constructed several PCSK9 variants with peptides truncated then overexpressed these truncated PCSK9 proteins in MARC-145 cells, followed by PRRSV strain HuN4 infection. The WB result showed that the C-terminal domain of PCSK9 was sufficient to inhibit PRRSV replication (Figure 2D).

### 3.3. PCSK9 Degrades PRRSV Receptor CD163 Through Lysosome Pathway

**Figure 3:**
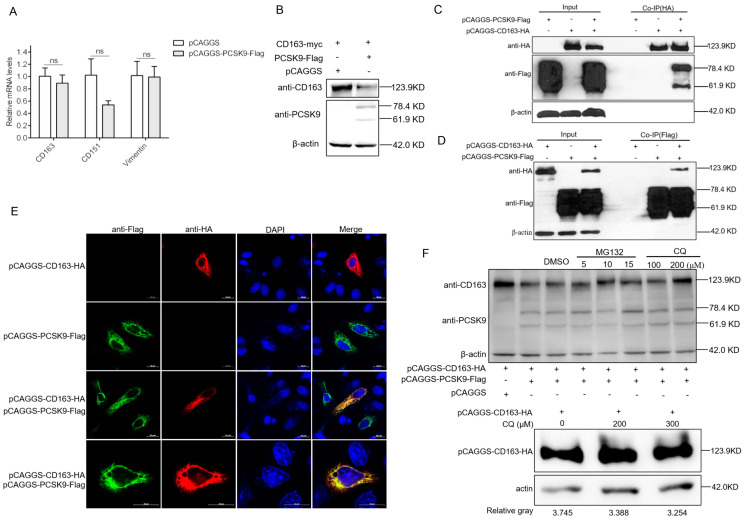
PCSK9 degrades the PRRSV receptor CD163 through the lysosome pathway (**A**) MARC-145 cells were transfected with either pCAGGS-PCSK9-flag or the empty vector pCAGGS. At 24 hpt, total RNAs were isolated and RT-qPCR was carried out to evaluate the relative expression of CD163, CD151, and vimentin. The mRNA expression levels were determined relative to GAPDH. Error bar: mean ± SEM; ns: no significant. (**B**) HEK-293T cells were transfected with pCAGGS-CD163-HA and pCAGGS-PCSK9-Flag or pCAGGS vector. The cell lysates were analyzed by WB for the PCSK9 protein and CD163 protein. (**C** and **D**) HEK-293T cells were cotransfected with different combinations of vectors as indicated. Cell lysates were harvested at 24 hpt, and immunoprecipitation was performed using antibodies against HA (**C**) or Flag (**D**), followed by WB analysis. Samples of input were included as controls. (**E**) HeLa cells were transfected with pCAGGS-PCSK9-flag and/or pCAGGS-CD163-HA. The cells were then fixed and permeabilized with 0.5% Triton X-100 for immunofluorescent staining with a mouse anti-HA antibody (red) and a rabbit anti-Flag antibody (green). Representative images are shown. (**F**). Top panel: HEK-293T cells were transfected with pCAGGS-PCSK9-Flag and/or pCAGGS-CD163-HA as indicated. At 18 hpt, the cells were further treated with/without the proteasome inhibitor MG132 or the lysosomal inhibitor chloroquine (CQ) or the vehicle, DMSO, for 6 h. Then, cell lysates were collected and analyzed by WB for PCSK9 and CD163 expression. Bottom panel: HEK-293T cells were transfected with pCAGGS-CD163-HA only, and at 18 hpt, the cells were further treated with CQ or the vehicle, DMSO, for 6 h. Then, cell lysates were collected and analyzed by WB for CD163 expression.

The function of PCSK9 is the binding of specific cell surface receptors to bring them towards the intracellular lysosome degradation compartments [49,50]. In addition to reducing the receptors through the lysosome, PCSK9 also can directly interact with CD36 and target the receptor to lysosomes through a mechanism involving the proteasome [51]. These findings suggest that PCSK9 may inhibit PRRSV replication through regulating the virus receptor presenting on target cells through the lysosomal and/or proteasomal pathway. We tested the mRNA abundance of several receptors—CD163, CD151, and vimentin—upon PCSK9 overexpression in MARC-145 cells. As expected, none of these receptors showed a significant difference in terms of mRNA amounts after PCSK9 overexpression (Figure 3A), suggesting that PCSK9 could regulate CD163 post translation. Therefore, HEK-293T cells were transfected with pCAGGS-CD163-HA and pCAGGS-PCSK9-Flag or pCAGGS vector. The cell lysates were analyzed by WB. Compared to control, the level of CD163 significantly decreased in PCSK9-overexpressing cells (Figure 3B). To further examine whether the PCSK9 protein could bind to CD163, HEK-293T cells were co-transfected with pCAGGS-CD163-HA and/or pCAGGS-PCSK9-Flag. The cells were lysed for immunoprecipitation with an antibody against HA tag, followed by WB analysis with antibodies against Flag and HA tag. As shown in Figure 3C, the level of PCSK9 protein in the presence of CD163 was significantly higher than that of the empty vector, whereas there were similar levels of the PCSK9 protein in the input with or without CD163 protein. We further confirmed the interaction between PCSK9 and CD163 by immunoprecipitating with an antibody against Flag tag followed by WB analysis with antibodies against Flag and HA tag (Figure 3D). Furthermore, colocalization studies were conducted by co-transfecting HeLa cells with pCAGGS-CD163-HA and/or pCAGGS-PCSK9-Flag. Immunofluorescence analysis showed that PCSK9 and CD163 colocalized both in the cytoplasm and on the cell membrane (Figure 3E).

We speculated that PCSK9 could degrade CD163 protein through the proteasome or lysosome. For this purpose, we cotransfected HEK-293T cells with pCAGGS-CD163-HA and pCAGGS-PCSK9-Flag then treated the cells with DMSO or the proteasomal inhibitor MG132 or lysosomal inhibitor chloroquine (CQ). The cell lysates were analyzed by WB with antibodies against HA and Flag tag. For the proteasomal pathway, compared to the DMSO control, CD163 abundance slightly increased in the MG132-treated cells with different doses (Figure 3F). For the lysosomal pathway, the WB result showed that lysosome inhibition resulted in a higher level of the CD163 protein compared to that following DMSO treatment (Figure 3F). This finding indicates that the lysosome pathway involves the degradation of CD163 by the PCSK9 protein.

### 3.4. PCSK9 Promotes Interferon Production in a Dose-Dependent Manner

**Figure 4:**
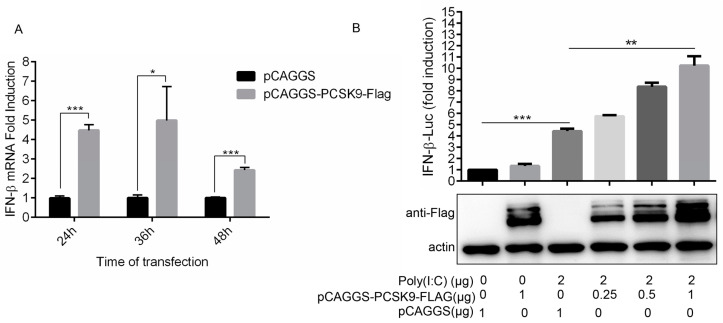
PCSK9 promotes interferon production in a dose-dependent manner. (**A**) MARC-145 cells were transfected with either pCAGGS-PCSK9-flag or the empty vector pCAGGS. At different time points as indicated after transfection, the cells were harvested and total RNAs were purified. The expression of IFN-β was assessed by RT-qPCR with specific primers for IFN-β. Error bar: mean ± SEM; *, *p* ≤ 0.05; ***, *p* ≤ 0.001. (**B**) HEK-293T cells were transfected with different amounts of pCAGGS-PCSK9-flag or the empty vector pCAGGS then treated with or without Poly (I:C). The activity of luciferase was monitored to evaluate the activity of the IFN-β promoter.

It has been reported that PCSK9 decreases IFN-β promoter/enhancer activity to suppress IFN-β production in humans [52]. To confirm whether the porcine PCSK9 could regulate the innate immunity response, we tested the effect of porcine PCSK9 on IFN-β production in MARC-145 cells. Unlike the effect of PCSK9 on IFN-β production in human cells, the qPCR result showed that PCSK9 promoted the production of IFN-β (Figure 4A). These contradictory results might be due to the different settings in the cell lines. To further investigate if PCSK9 could bind to the promoter region of the IFN-β gene and regulate the transcription of PCSK9, we measured IFN-β gene promoter activity in PCSK9-overexpressing HEK-293T cells. The result showed that PCSK9 enhanced the activity of the promoter of the IFN-β gene to regulate IFN-β transcription (Figure 4B).

### 3.5. PRRSV Down-Regulates PCSK9 Expression Both in MARC-145 Cells and in PAM Cells

**Figure 5:**
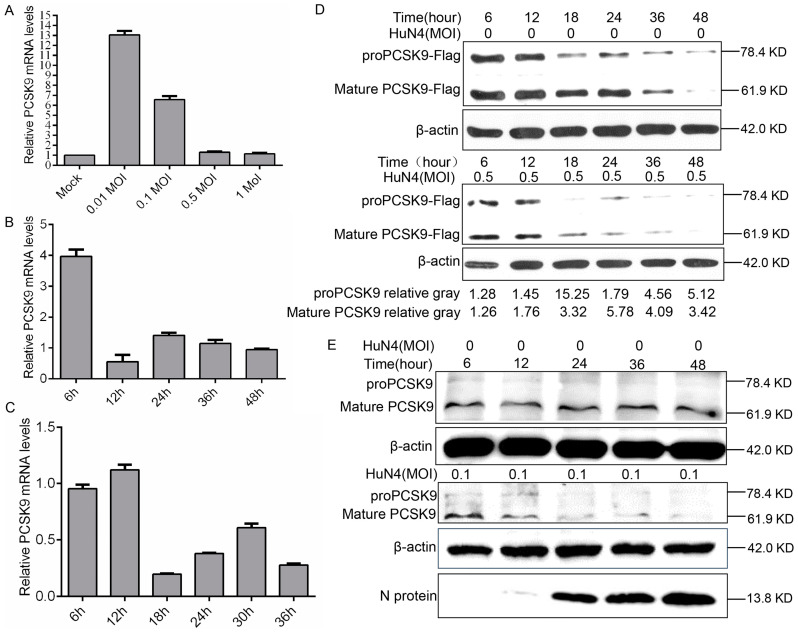
PRRSV down-regulates PCSK9 expression. (**A**) MARC-145 cells were infected with different doses of the PRRSV strain HuN4 (0.01, 0.1, 0.5, and 1 MOI). At 36 hpi, total RNAs were isolated and RT-qPCR was performed to evaluate the relative expression of PCSK9. Error bar: mean ± SEM. (**B**) MARC-145 cells were infected with the PRRSV strain HuN4 (MOI = 0.1). Total RNAs were isolated at the time points as indicated, and the relative expression levels of PCSK9 were determined by RT-qPCR. (**C**) Porcine alveolar macrophages (PAMs) were infected with the PRRSV strain HuN4 (MOI = 0.5). Total RNAs were purified at the time points as indicated, and PCSK9 expression levels were determined by RT-qPCR. (**D**) MARC-145 cells were transfected with the pCAGGS-PCSK9-Flag vector. The cells were infected with the HuN4 virus (MOI = 0.5) or DMEM as control at 36 hpt. Cell lysates were collected and analyzed by WB to assess PCSK9 expression. The values below were calculated from pre-PCSK9 or mature PCSK9 gray values against the corresponding β-actin gray values, then the values from the HuN4-infected cells were normalized to the corresponding values from the HuN4-uninfected cells. (**E**) PAMs were infected with the HuN4 virus at a MOI of 0.1 or DMEM as a control. Cell lysates were collected and analyzed by WB to assess endogenous PCSK9 expression and PRRSV N protein expression.

To examine the possible influences of PRRSV on PCSK9 expression, we infected MARC-145 cells with different doses of the PRRSV strain HuN4. The RT-qPCR results showed that endogenous PCSK9 mRNA expression increased significantly compared to that in the mock control when the cells were infected with a low dose of the virus (Figure 5A). Interestingly, the PCSK9 expression level is comparable to that in the mock control when they are infected with a high dose of the virus (Figure 5A). To further examine the PCSK9 expression changes during PRRSV infection, we infected MARC-145 cells with HuN4 and quantified PCSK9 expression using RT-qPCR. The PCSK9 expression level decreased as the time increased after infection (Figure 5B). We confirmed this finding with PAM cells; as shown in Figure 5C, PRRSV reduces the PCSK9 expression level in a time-dependent manner. We further examined the PCSK9 protein level at various time points in MARC-145 cells that were transfected with PCSK9 followed by HuN4 virus infection. The WB result showed that the PCSK9 protein level decreased as the infection time increased compared to that in the mock infection control (Figure 5D). A similar result was observed in PAMs; endogenous PCSK9 expression decreased as the infection time increased, whereas that of the PRRSV N protein did the opposite (Figure 5E).

### 3.6. PRRSV nsp11 Negatively Regulates PCSK9 Expression

**Figure 6:**
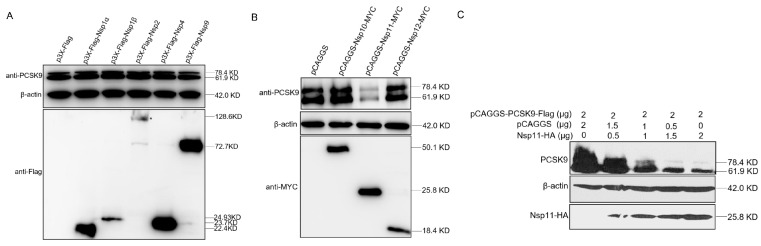
PRRSV nsp11 could inhibit PCSK9 expression. (**A** and **B**) PRRSV non-structural protein sequences including nsp1α, nsp1β, nsp2, nsp4, nsp9, nsp10, nsp11, and nsp12 were cloned into p3X-Flag or pCAGGS vectors with Flag or MYC tag. HEK-293T cells were cotransfected with pCAGGS-PCSK9-Flag (2 μg) and different nsp constructs (2 μg). At 36 hpt, cell lysates were collected and analyzed by WB for PCSK9 expression and nsp expression. (**C**) PCSK9 expression construct pCAGGS-PCSK9-Flag was cotransfected with different doses of an expression vector encoding nsp11. At 36 hpt, cell lysates were collected and analyzed by Western blotting.

PCSK9 expression increased in the early stage of PRRSV infection, but the increase was suppressed in the late stages of infection, suggesting that proteins produced during PRRSV replication could negatively regulate PCSK9 expression. To explore which nsps of PRRSV were involved in the down-regulation of PCSK9 expression, several nsps including nsp1α, nsp1β, nsp2, nsp4, nsp9, nsp10, nsp11, and nsp12 of PRRSV were cloned and cotransfected with pCAGGS-PCSK9-Flag into HEK-293T cells. At 24 hpt, cell lysates were collected and analyzed by WB. The PCSK9 expression level was significantly decreased by PRRSV nsp11, whereas the effects of other PRRSV nsps on PCSK9 expression were not obvious (Figure 6A,B). Moreover, we cotransfected HEK-293T cells with different amounts of pCAGGS-NSP11-HA and the same amount of the pCAGGS-PCSK9-Flag plasmid. We found that nsp11 down-regulated PCSK9 expression in a dose-dependent manner (Figure 6C). These data suggest that nsp11 could antagonize the antiviral function of PCSK9 during PRRSV replication.

### 3.7. PRRSV nsp11 Inhibits PCSK9 via Its Endoribonuclease Activity

**Figure 7:**
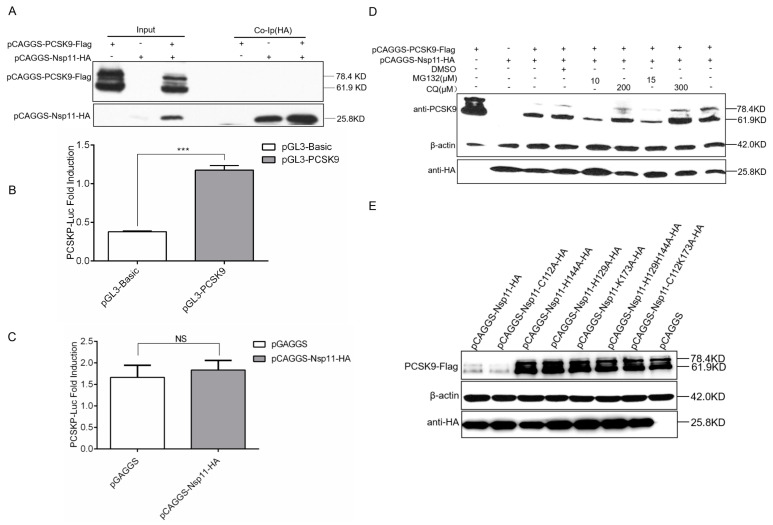
PRRSV nsp11 inhibits PCSK9 expression through its endoribonuclease activity. (**A**) HEK-293T cells were cotransfected with different combinations of vectors as indicated. Cell lysates were harvested at 24 hpt, and immunoprecipitation was performed using an antibody against HA, followed by WB analysis. (**B** and **C**) 855 bp PCSK9 promoter sequence was cloned into the pGL3-Basic vector. pGL3-PCSK9 was cotransfected with pCAGGS-HA-Nsp11(1 μg) or pCAGGS empty vector (1 μg) as a negative control into HEK-293T cells. Error bar: mean ± SEM; ***, *p* ≤ 0.001.; ns: no significant. (**D**) HEK-293T cells were transfected with pCAGGS-PCSK9-Flag (2 μg) and/or pCAGGS-HA-Nsp11 (2 μg) as indicated. At 18 hpt, the cells were further treated with/without the proteasome inhibitor MG132 or lysosomal inhibitor chloroquine (CQ) or vehicle, DMSO, for 6 h. Then, cell lysates were collected and analyzed by WB to assess PCSK9 and nsp11 expression. (**E**) A set of nsp11 constructs containing mutations that could inactivate endoribonuclease activity (nsp11-H129A, nsp11-H144A, nsp11-K173A, nsp11-H129H144A, and nsp11-C112K173A) or deubiquitinating activity (nsp11-C112A) (2 μg) were generated. pCAGGS-PCSK9-Flag (2 μg) was cotransfected with nsp11 mutants and wild type nsp11 into HEK-293T cells. WB was performed to analyze PCSK9 and nsp11 expression.

In light of the significant consequences of the nsp11-mediated negative regulation of PCSK9 expression, we further explored the molecular mechanism. Firstly, to examine whether there was an interaction between the nsp11 and PCSK9 proteins, cells were co-expressed with pCAGGS-HA-Nsp11 and pCAGGS-PCSK9-Flag. Co-IP was performed with an antibody against HA, followed by WB analysis. As shown in Figure 7A, nsp11 did not interact with the PCSK9 protein. Secondly, to figure out the binding of nsp11 to the promoter regions of PCSK9 to inhibit PCSK9 transcription, we measured PCSK9 promoter activity in nsp11-over-expressing cells. For this purpose, HEK-239T cells were cotransfected with a luciferase reporter containing the PCSK9 promoter with or without pCAGGS-HA-Nsp11. The reporter luciferase activity showed no significant difference between the nsp11-over-expressing cells and the control group (Figure 7B,C), indicating that nsp11 could not regulate PCSK9 transcription by binding to the PCSK9 promoter. Then, we investigated if nsp11 targeted PCSK9 through the proteasome pathway or lysosome pathway. Through the inhibition of the proteasome or lysosome, we confirmed that neither the proteasome nor lysosome was responsible for the down-regulation of PCSK9 (Figure 7D). Finally, we constructed a set of nsp11 constructs containing mutations that could inactivate either the endoribonuclease activity or deubiquitinating activity of nsp11 and cotransfected pCAGGS-PCSK9-Flag with nsp11 mutants into cells. The WB results showed that nsp11 mutants with inactivated endoribonuclease activity lost the function of antagonizing PCSK9 antiviral activity, whereas those nsp11 mutants that lost their deubiquitinating activity did not (Figure 7E). Collectively, these results showed that nsp11 negatively regulated PCSK9 expression through its endoribonuclease activity, not deubiquitinating activity.

## 4. Discussion

Viruses regulate host cellular components and try to take control of the normal cell networks to facilitate their survival and replication. Conversely, the cell hosts are responsive to viral infection and fight viruses through a variety of anti-viral approaches. Although the interactions between PRRSV and cells have been extensively studied [2], more research on host changes upon PRRSV infection is needed, because the knowledge of these factors is essential for understanding viral infection and can offer potential targets for antiviral therapies as well as a new insight for vaccine design. In this study, we found that a new antiviral factor PCSK9 could significantly inhibit the replication of PRRSV. We also explored the amino acid of porcine PCSK9 key for PCSK9 maturation and the effect of pre-PCSK9 and mature PCSK9 on PRRSV replication. Intriguingly, both the pre-PCSK9 and mature PCSK9 show antiviral activity against PRRSV. In this case, we hypothesized that a specific domain of PCSK9 instead of whole protein could play an important role in the inhibition of PRRSV replication. Further investigation indicated that the C-terminal domain of PCSK9 has antiviral activity.

Several host factors have been discovered that can suppress PRRSV replication, most of them, by targeting the innate immune pathways [2,53]. As for the role of PCSK9, studies suggest that PCSK9 plays a role in immunity modulation, such as by influencing IFN and inflammatory factor production. Our results showed that PCSK9 could increase the mRNA level of IFN-β by influencing its promoter. This finding suggests that PCSK9 may inhibit PRRSV replication in part through up-regulation of the IFN-β product. However, this finding is not in line with the PCSK9 function reported in the previous study, in which IFN-β expression was inhibited in human cells [52]. In the previous study, PCSK9 suppressed IFN-β expression through inhibiting activating transcription factor-2 (ATF-2)/c-Jun dimerization and the binding of ATF-2/c-Jun to the IFN-β enhancer via interaction with ATF-2. However, we showed that PCSK9 increased IFN-β expression by influencing its promoter. The mechanism underlying PCSK9’s effect on IFN-β expression needs to be further studied.

PCSK9 can impede HCV replication in human live cells by decreasing the expression of LDLR through binding to LDLR and delivering the PCSK9–LDLR complex to lysosomes for degradation [49,50]. In addition to targeting cell receptors through the lysosomal pathway, evidence shows that PCSK9 can partially target protein degradation through the proteasomal pathway. One study shows that PCSK9 reduces the expression of the CD36 receptor by directly interacting with CD36 and targeting the receptor to lysosomes through a mechanism involving the proteasome [51]. Based on these findings, we probe the effect of PCSK9 on CD163, which is required for PRRSV infection. Similar to CD36 or LDLR degradation by PCSK9 via the lysosome, we showed that PCSK9 could bind to CD163 and decrease the CD163 protein level. These observations imply that PCSK9 inhibited PRRSV replication by delivering the PCSK9–CD163 complex to lysosomes for degradation. However, we cannot rule out other mechanisms of PCSK9’s effect on PRRSV replication. The possible pathway underlying the regulation by PCSK9 of PRRSV replication needs to be addressed further.

PRRSV nsp11 possesses both endoribonuclease activity and deubiquitinating activity. The endoribonuclease activity of nsp11 is conserved and unique for viruses in the order Nidovirales. The NendoU domain of PRRSV nsp11 elicits the nuclease activity. PRRSV nsp11 is an IFN antagonist, and the endoribonuclease activity is critical for IFN suppression [53]. PRRSV nsp11 also can target MAVS and degrade the MAVS mRNA, leading to the failure of the activation of downstream pathways to suppress IFN production [19]. Unlike previous findings, we show that nsp11 antagonizes PCSK9’s antiviral activity by inducing the degradation of the PCSK9 transcript through its endoribonuclease activity. However, the endoribonuclease activity of nsp11 is not specific. We also confirmed this by overexpressing EGFP and nsp11. Consistent with the previous findings [54], nsp11 could slightly decrease the expression of EGFP (data not shown). The mechanism by which nsp11 selectively degrades PCSK9 RNA needs to be further investigated.

In summary, we have investigated the relationship between PCSK9 and PRRSV replication and revealed that PCSK9 has antiviral activity against PRRSV. The C-terminal domain of PCSK9 plays a central role in its antiviral activity. PCSK9 could bind to CD163 and deliver CD163 to the lysosome for degradation. Besides, we reveal that PRRSV can antagonize the antiviral activity of PCSK9 through nsp11 endoribonuclease activity. Our finding broadens our understanding of how PRRSV nsp11 antagonizes host factors to facilitate viral survival and replication, which provide further insights into the interaction between PRRSV and the cell host, and offer a new antiviral target for curbing the spread of PRRSV.

## Figures and Tables

**Table 1:** Primers used in this study.

Primer ^a^	Sequence (5′-3′) ^b^	Usage
Mature-PCSK9-F	TGCTCTAGAATGAGCATCCCGTGGAACCTGGAG	Amplification of mature PCSK9 (151–690aa)
Mature-PCSK9-R	CCGGAATTCTCACTTATCGTCGTCATCCTTGTAATCCTGGGACTCCTGGGAGGCCTC	
PCSK9-151-400-Flag-F	TCATTTTGGCAAAGATGAGCATCCCGTGGAACCTGG	Amplification of PCSK9 catalysis domain (151–400aa)
PCSK9-151-400-Flag-R	CCAGATCTGAATTTTACTTATCGTCGTCATCCTTGTAATCCGGCTCAGCCGTCAGCATC	
PCSK9-448-690-Flag-F	TCATTTTGGCAAAGATGGGTGGGCAGCTGTTCTGCA	Amplification of PCSK9 C-terminal domain (448–690aa)
PCSK9-448-690-Flag-R	TACCAGATCTGAATTTCACTTATCGTCGTCATCCTTGTAATCCTGGGACTCCTGGGAG	
PCSK9-Q150A-F	CGTCTTTGCGGCGAGCATCCCGTGGAACCTGGAGCGG	Amplification of mutation PCSK9(Q150A)
PCSK9-Q150A-R	CGGGATGCTCGCCGCAAAGACGAAGGAGTCCTCCTCG	
PCSK9-D182A-F	GTATCTCTTAGCCACCAGCATCCAAAGTGGCCACC	Amplification of mutation PCSK9 (D182A)
PCSK9-D182A-R	GGATGCTGGTGGCTAAGAGATACACCTCCACCAGG	
PCSK9-H222A-F	GTGTGACAGCGCCGGCACCCACCTGGCCGGGGT	Amplification of mutation PCSK9 (H222A)
PCSK9-H222A-R	GTGGGTGCCGGCGCTGTCACACTTGTTCGCCTG	
PCSK9-N313A-F	CCGCTGCTGGCGCCTTCCGGGACGACGCCTGCCTC	Amplification of mutation PCSK9 (N313A)
PCSK9-N313A-R	CGTCCCGGAAGGCGCCAGCAGCGGCCACCAGCACT	
PCSK9-S382A-F	CAGAGCGGGACGGCACAGGCTGCCGCCCATGTGGC	Amplification of mutation PCSK9 (S382A)
PCSK9-S382A-R	CGGCAGCCTGTGCCGTCCCGCTCTGCGACGTGAAGC	
PCSK9-N529A-F	CTGCCCCGGGCCGCCTGCAGCATCCACATGGCTCCA	Amplification of mutation PCSK9 (N529A)
PCSK9-N529A-R	TGGATGCTGCAGGCGGCCCGGGGCAGCAGGCAGCAC	
pGL3-PCSK9-F	CGGGGTACCTTGGCTGGTTGGTGAGGTGAG	Amplification of PCSK9 promoter
pGL3-PCSK9-R	CCGCTCGAGGCAGCAGTAGCAGCAGCGGCGGC	
Nsp11-C112A-F	GAGGTAGATGCTCGAGAGTATCTTGATGATCGGGAGC	Amplification of Nsp11 (C112A, H112K173A)
Nsp11-C112A-R	TACTCTCGAGCATCTACCTCAATTCGGCCGGTGCTGAAG	
NSP11-H129A-F	GTCCCTCCCAGCTGCCTTCATCGGCGATGTCAAAG	Amplification of Nsp11 (H129A, H129H144A, H129K173A)
NSP11-H129A-R	GATGAAGGCAGCTGGGAGGGACTCAGCAACCTCTC	
Nsp11-H144A-F	GTTGGGGGATGTGCTCACGTTACCTCCAAATACCTTC	Amplification of Nsp11 (H144A, H129H144A)
Nsp11-H144A-R	GAGGTAACGTGAGCACATCCCCCAACGGTGGTACCT	
Nsp11-K173A-F	GAAAGCCGCGGCAGCAGTTTGCACATTGACGGATGTGTAC	Amplification of Nsp11 (K173A, H112K173A, H129K173A)
Nsp11-K173A-R	GCAAACTGCTGCCGCGGCTTTCCCGGGGCTCGAAACCCCG	
EGFP-Flag-F	TCATTTTGGCAAAGATGGTGAGCAAGGGCGAGG	Amplification of EGFP-Flag
EGFP-Flag-R	TACCAGATCTGAATTTTACTTATCGTCGTCATCCTTGTAATCCTTGTACAGCTCGTCC	
q-Pig-PCSK9-F	CCACGTCCTCACAGGTTGC	qPCR for detection of pig PCSK9
q-Pig-PCSK9-R	CGTGGACACTGGCCTTCTC	
q-Monkey-PCSK9-F	ACCCGTGTCCACTGCCATCAG	qPCR for detection of monkey PCSK9
q-Monkey-PCSK9-R	ACCTCGTGGCCTCAGCACAG	
human-PCSK9-F	GAAGATGAGTGGCGACCT	qPCR for detection of human PCSK9
human-PCSK9-R	CCGGTGGTCACTCTGTATGCT	
Pig-GAPDH-F	ATGGTGAAGGTCGGAGTGAAC	qPCR for detection of human GAPDH
Pig-GAPDH-R	CGTGGGTGGAATCATACTGG	
Monkey-GAPDH-F	CCTTCCGTGTCCCTACTGCCAAC	qPCR for detection of monkey GAPDH
Monkey-GAPDH-R	GACGCCTGCTTCACCACCTTCT	
PRRSV-N-F	AAAACCAGTCCAGAGGCAAG	qPCR for detection of PRRSV N
PRRSV-N-R	CGGATCAGACGCACAGTATG	
Monkey-IFN-β-F	GCAATTGAATGGAAGGCTTGA	qPCR for detection of monkey IFN-β
Monkey-IFN-β-R	CAGCGTCCTCCTTCTGGAACT	
pIFN-β-F	GGCGGTACCCTTGGCTTATGGTGGTTTTTTTTG	Amplification of PCSK9 promoter
pIFN-β-R	TTTCTCGAGGCTCCACTACTCAAGTGCTGAAG	

^a^ F denotes forward PCR primer; R denotes reverse PCR primer. ^b^ Restriction sites, mutated nucleotides, and homologous arm are underlined.
